# Nonlinear compression toward high-energy single-cycle pulses by cascaded focus and compression

**DOI:** 10.1126/sciadv.abo1945

**Published:** 2022-08-03

**Authors:** Ming-Shian Tsai, An-Yuan Liang, Chia-Lun Tsai, Po-Wei Lai, Ming-Wei Lin, Ming-Chang Chen

**Affiliations:** ^1^ Institute of Photonics Technologies, National Tsing Hua University, Hsinchu 300044, Taiwan.; ^2^ Institute of Nuclear Engineering and Science, National Tsing Hua University, Hsinchu 300044, Taiwan.; ^3^ Department of Physics, National Tsing Hua University, Hsinchu 300044, Taiwan.; ^4^ Frontier Research Center on Fundamental and Applied Sciences of Matters, National Tsing Hua University, Hsinchu, Taiwan.

## Abstract

The advancement of contemporary ultrafast science requires reliable sources to provide high-energy few-cycle light pulses. Through experiments and simulations, we demonstrate an arrangement of pulse postcompression, referred to as cascaded focus and compression (CASCADE), for generating millijoule-level, single-cycle pulses in a compact fashion. CASCADE is realized by a series of foci in matter, whereas pulse compression is provided immediately after each focus to maintain a high efficiency of spectral broadening. By implementing four stages of CASCADE in argon cells, we achieve 50-fold compression of millijoule-level pulses at 1030 nanometers from 157 to 3.1 femtoseconds, with an output pulse energy of 0.98 millijoules and a transmission efficiency of 73%. When driving high harmonic generation, these single-cycle pulses enable the creation of a carrier-envelope phase-dependent extreme ultraviolet continuum with energies extending up to 180 electron volts, providing isolated attosecond pulses at the output.

## Teaser

A postcompression arrangement was introduced to efficiently shorten the pulses into the single-cycle regime.

## INTRODUCTION

Ultrafast pulses have improved our understanding of how atomic, electronic, and magnetic structures move and change over their fundamental time scales. Attosecond light pulses, particularly those driven by mid–infrared (IR) pulses via high-order harmonic generation (HHG), are receiving substantial attention (*1*) because they provide further insight into how chemical bonds break and form (*2*), how light waves drive devices into the petahertz regime (*3*–*6*), and how excited electrons reshape the energy landscape of material transformations (*7*, *8*). To efficiently produce an isolated attosecond pulse, intense single-cycle pulses become essential, which prevent the HHG process from repeating itself every half cycle, resulting in an attosecond pulse train.

Clever schemes have been devised for intense few-cycle pulse generation, following three main approaches: broad bandwidth amplifiers, optical waveform synthesizers, and nonlinear postcompression techniques. In the first approach, broadband seed pulses can be directly amplified without gain narrowing, including, for example, optical parametric (chirped pulse) amplification. A broad phase-matching bandwidth is allowed in unique nonlinear crystals that operate at specific wavelengths. Two- or three-cycle pulses were demonstrated at a central wavelength of 850 nm using Beta barium borate (BBO) (*9*) and 1550 nm using Bismuth Borate (BIBO) (*10*). Regarding the waveform synthesizer, subcycle pulses can be realized by coherently superposing light waves of different colors, generated by optical parametric (chirped-pulse) amplification (*11*) or supercontinuum generation (*12*). Monitoring and active stabilization of all relative time delays, relative phases, and carrier-envelope phases (CEPs) are essential for realizing a shot-to-shot stable synthesized electric field. However, because of the sophisticated technology required, very few laboratories have been able to produce few-cycle pulses and gain access to attosecond dynamics.

A relatively straightforward strategy is nonlinear compression (*13*, *14*), which is based on a nonlinear interaction, typically self-phase modulation, to increase the spectral bandwidth, where the pulse chirp can be removed by dispersion compensation to shorten the pulse duration. Currently, three arrangements can produce millijoule-level few-cycle pulses: a hollow core waveguide (*15*–*17*), multiple thin plates (*18*–*21*), and multipass cells (*22*, *23*). The hollow core waveguide technique uses a hollow waveguide filled with gases as a nonlinear medium to expand the spectral bandwidth of the driving lasers. The output mode quality is well behaved, with *M*^2^ values lower than 1.2, while the power transmission is typically 70%. To achieve a high compression ratio, several meter-long high-quality capillaries or multistage arrangements are required. A multiple-plate continuum, using a sequence of thin plates as nonlinear media, shows great potential for few-cycle pulse generation. This technique is compact, cost-effective, and less sensitive to beam pointing. However, this approach typically yields a worse beam quality than the hollow waveguide. Meanwhile, damage to plates and degradation in intrapulse coherence could be an issue when using millijoule-level lasers (*24*). The multipass gas cell technique is based on a Herriot cavity. A few- to single-cycle pulse generation is quite promising once the cavity mirror could keep high reflectivity and broad spectral bandwidth.

In this work, we propose an arrangement of postcompression technique that can efficiently compress the pulse to the single-cycle regime. We refer to this as cascaded focus and compression (CASCADE). One CASCADE unit comprises a focus in matter to carry out nonlinear broadening, together with one compressor that shortens the pulse duration. When using only four-unit CASCADE in Ar, we realized the compression of millijoule-level 1030-nm pulses from 157 to 3.1 fs (single cycle) in full width at half maximum (FWHM) with a good transmission efficiency of 73%. The experimental spectra show quantitatively good agreement with those acquired from three-dimensional numerical simulations, which indicates that both self-phase modulation and ionization help spectral broadening behind CASCADE. In addition, a satisfactory homogeneity of spectral broadening up to 93.5% across the beam profile was verified when compared to the ideal value of 95.6% acquired from the simulation. Briefly, CASCADE exhibits the advantages of simplicity, high compression efficiency, and multistage scalability. Last, HHG driven by one-cycle CASCADE pulses was performed. We observed a highly CEP-dependent Extreme ultraviolet (EUV) continuum. The attosecond streaking reveals that the CASCADE pulse from a compact and efficient Yb laser is able to provide isolated attosecond pulses via HHG. We believe that this single-cycle postcompression technique would significantly facilitate time-resolved studies and has an immediate impact on both fundamental and applied aspects of strong-field physics and attosecond science.

## RESULTS

**Fig. 1.**
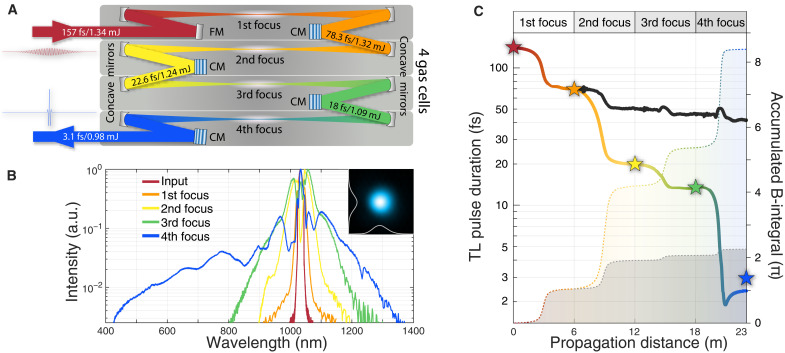
Schematic and spectra of a four-unit CASCADE. (**A**) Nonlinear spectral broadening is realized through a series of foci in four Ar-filled cells. Pulse compression is applied immediately after every focus. CM is the set of chirped mirrors as the pulse compressor. FM is a flat mirror. Because the beam propagation in CASCADE can be folded, we have packaged this four-unit CASCADE into a compact 3 m–by–0.3 m–by–0.3 m box. (**B**) Power spectra of the input pulse and the output pulse generated after every focus. The inset shows the far-field beam profile of the single-cycle pulse. a.u., arbitrary units. (**C**) Numerical simulations for estimating the transform-limited (TL) pulse duration (solid lines) and the accumulated B-integral (filled areas with dotted outline) versus the propagation distance in a series of foci with (color gradient) and without (black) compression between foci. Solid stars show the TL pulses based on the experimentally observed spectrum in (B).

The experimental setup starts with one CEP-stabilized Yb-KGW laser (Light-Conversion/Pharos), delivering 1.34-mJ pulses at a central wavelength of 1030 nm and at a repetition rate of 4 kHz. The pulse duration was 157 fs, which corresponds to an input peak power of 8.5 GW. A schematic of the four-unit CASCADE experiment is shown in Fig. 1A. One CASCADE unit contained a focus in an Ar-filled cell, together with one set of chirp mirrors (CMs) used as the pulse compressor. The broadening bandwidth and output beam quality can be optimized by tuning the pressure in each gas cell. In the first CASCADE unit, the 157-fs pulse achieves nonlinear spectral broadening by focusing on a 760-torr Ar cell and then compressing down to 78.3 fs through two pieces of CM#1, whose dispersion details are listed in Table 1. The following second (third and fourth) unit is filled with 760 torr (210 and 150 torr) of Ar, while the resulting pulse is compressed by 1× CM#1 (1× CM#2 and 3× CM#2 and a pair of fused silica wedges), further shortening the pulses down to 22.6 fs (18 and 3.1 fs). Both CM#1 and CM#2 were customized by UltraFast Innovations. The nonlinear compression efficiency of the first (second, third, and fourth) unit of CASCADE, including all concave mirrors, flat mirrors, and CMs, was 98% (98, 90.5, and 90%) individually. Overall, there is an additional 5% energy loss caused by the folding mirrors between units. Millijoule-level single-cycle laser pulses at a wavelength of 1 μm have been successfully generated using CASCADE with a transmission efficiency of 73%. The experimental parameters of CASCADE are listed in Table 2. Figure 1B shows the supercontinuum generated after each focus. After the fourth focus in CASCADE, spectral content down to the −25-dB level was generated in the span from 420 to 1360 nm. Long-term power behavior also showed excellent stability with a 0.314% deviation (see fig. S7). The spatial and temporal details of the resulting pulses are described in the following paragraphs:

**Table 1.** Chirped mirrors used in CASCADE. GDD, group delay dispersion; TOD, third-order dispersion; FOD, fourth-order dispersion; FID, fifth-order dispersion.

	**Range (nm)**	**GDD (fs^2^)**	**TOD (fs^3^)**	**FOD (fs^4^)**	**FID (fs^5^)**
CM#1	950–1100	−437	2180		
CM#2	595–1200	−231	−445	1849	−3490

**Table 2.** Experimental parameters in CASCADE. CM is chirped mirrors whose dispersion detail is listed in Table 1. FS is a pair of fused silica wedges.

**Unit**	**Input pulse energy (mJ)**	**Output pulse energy (mJ)**	**Focal length (m)**	**Ar pressure (torr)**	**Compressor**	**Throughput (%)**
First	1.342	1.315	3	760	2 × CM#1	98
Second	1.315	1.288	3	760	1 × CM#1	98
Third	1.238	1.12	3	210	1 × CM#2	90.5
Fourth	1.088	0.988	2.5	150	3× CM#2 + FS	90

Particular attention has been paid to pulse compression, which is immediately applied after every focus in CASCADE. This is understandable, because the B-integral ∆φ_NL_(*t*) is expressed as

$$∆\varphi_{\text{NL}}(t)=-\frac{\omega_{0}}{c}n_{2}\int_{0}^{L}I(t,z)\mathrm{dz}≅-\frac{\omega_{0}}{c}n_{2}I(t)L$$ (1)

where ω_0_ is the angular frequency of the driving pulses, *c* is the speed of light, *n*_2_ is the nonlinear Kerr coefficient of the medium, *L* is the propagation distance at the focus, and *I*(*t*, *z*) is the laser profile. In the last term, we assumed that the pulse close to the focus had a similar temporal profile. The instantaneous frequency ω_inst_(*t*) is expressed as

$$\omega_{\text{inst}}(t)=\frac{d}{\mathrm{dt}}[\omega_{0}t+∆\varphi_{\text{NL}}(t)]=\omega_{0}-\frac{\omega_{0}}{c}n_{2}\frac{\mathrm{dI}(t)}{\mathrm{dt}}L$$ (2)

This indicates that the instantaneous frequency generated is proportional to the intensity slope of the driving pulse, *dI*(*t*)/*dt*. Shorter pulses, which yield a larger *dI*(*t*)/*dt*, can broaden the frequency more efficiently. Note that once there is laser-induced ionization, the additional change in refraction index caused by the plasma should be included in the B-integral, ∆φ_NL_(*t*).

Further insight into the nonlinear process occurring in each CASCADE unit is acquired by conducting a three-dimensional beam propagation numerical simulation (see section S1), which validates the effect of spectral broadening and estimates the accumulated B-integral. Figure 1C shows the accumulated B-integral and the corresponding transform-limited (TL) pulse duration versus propagation distance. When the pulse compression is provided immediately after each spectral broadening at the focus, the B-integral increases by 1.07π rad (3.15π, 1.17π, and 3π rad), while the TL pulse duration becomes 68 fs (19.6, 13.2, and 2.4 fs) after the first (second, third, and fourth) focus. A total B-integral of 8.4π rad was accumulated in this four-unit CASCADE. The TL values estimated by our numerical simulation were in good agreement with those obtained by taking the Fourier transform of the measured spectra shown in Fig. 1B. In contrast, when multifocusing is carried out without any dispersion engineering between foci, simulations show that the accumulated B-integral increases slowly to a saturation value of 2.2π rad, and the spectral broadening after four foci is limited to a much longer TL of 41.5 fs, which is ≈17 times longer than that acquired from the CASCADE scheme. The comparison shown in Fig. 1C thus illustrates the essence of conducting compression in CASCADE, which allows the nonlinear phase to accumulate in a laser pulse with a much greater efficiency than that in the uncompressed case under the same propagation distance. As the operation of CASCADE minimizes the number of mirror reflections for a pulse to achieve a sufficiently broad spectrum, it facilitates the achievement of a high transmission efficiency and sustains a wide bandwidth in postcompression; otherwise, the limited reflectivity of optics can constantly reduce the energy and bandwidth of the output pulse with successive reflections. Therefore, CASCADE significantly facilitates a few- or single-cycle pulse generation with an excellent postcompression efficiency, as evident from the results summarized in Table 2.

**Fig. 2.**
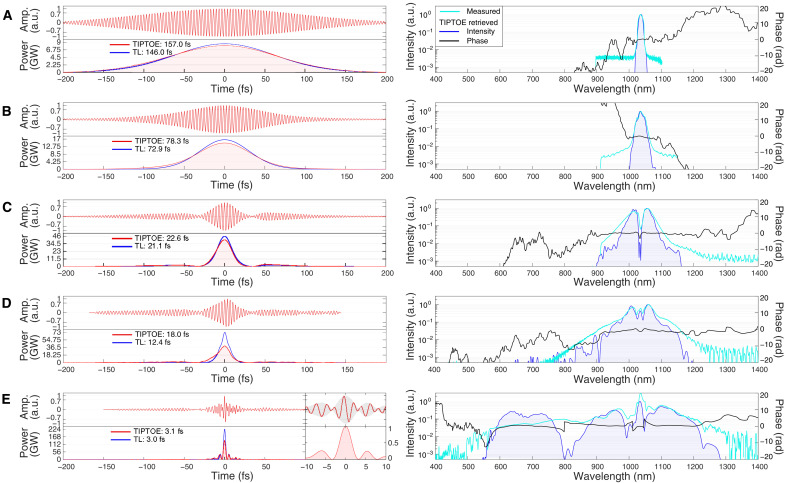
Temporal characterization of the postcompressed pulses. The waveform of (**A**) the input pulse and the (**B**) first, (**C**) second, (**D**) third, and (**E**) fourth CASCADE characterized by TIPTOE in air. The left column presents the measured electric field amplitude (red lines) and the corresponding intensity profile (red areas), together with the TL intensity profile (blue lines). The inset in (**E**) shows a zoomed-in region near the center of the temporal profile. The right column presents the spectrum (blue areas) and the spectral phase (black line), which are obtained by taking the Fourier transform of the measured waveform from the left column. For comparison, the independently measured spectra using a Si-based spectrometer (either ASEQ or Ocean HR4000) and an InGaAs-based spectrometer (BWtek Sol 1.7) are shown by the cyan lines.

The left column in Fig. 2 shows the resulting pulse in CASCADE, which is carefully characterized by tunneling ionization with a perturbation for the time-domain observation of an electric field (TIPTOE) (*25*–*27*), because this is one of the most straightforward and unambiguous ways to examine the electric field waveform of a light field with subfemtosecond temporal resolution (for more details about the TIPTOE, see Materials and Methods and section S2). The right column in Fig. 2 shows the corresponding spectra and spectral phases, which were obtained by directly taking the Fourier transform of the measured waveform in the left column. It is evident that in the first unit of CASCADE, no satellite pulse is observed in the temporal domain. An energy ratio up to 99.3% was contained in the main pulse, which was derived from a Gaussian fit. In the second unit of CASCADE, owing to the sharp amplitude and phase modulation at 1030 nm, two weak satellite pulses appear ≅60 fs ahead and behind the main pulse, resulting in an energy ratio decrease to 76.2% in the main pulse. These incompressible satellite pulses can be viewed as main pulse losses. That is, the degradation of the energy ratio in the main pulse is attributed to the incompressible satellite pulses left behind by postcompression. After the following two units of CASCADE, the amplitude and phase jump close to 1018 and 800 nm further decrease the energy ratio in the main pulse to 45.4%. Nevertheless, the measured 3.1-fs pulse width is close to the TL duration and has very low side satellite pulses of 25% relative intensity, appearing at ±5.3 fs. Considering the transmission efficiency of CASCADE and partial energy in satellite pulses, the peak power of the pulses was boosted 17 times from 8.5 to 145 GW.

**Fig. 3.**
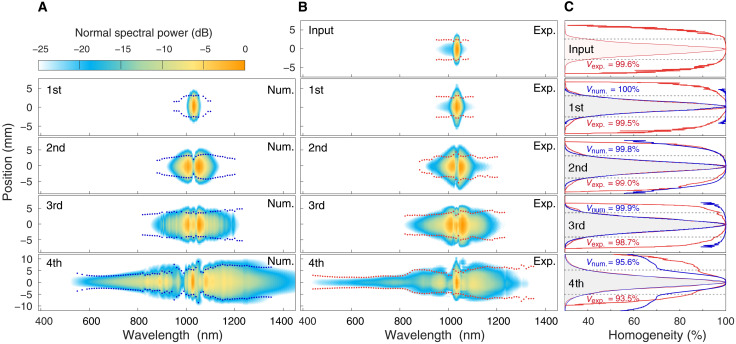
Beam quality of the postcompressed pulses. The comparison of the spatial-spectral distribution of the postcompressed beam along the transverse direction between numerical simulations (**A**) and experimental measurements (**B**). (**C**) Spatiospectral beam homogeneity and normalized integrated power calculated from (A) and (B). Dotted lines in (A) to (C) indicate the beam waist at 1/e^2^ level.

Moreover, the spatial-spectral homogeneity was measured to quantify the postcompressed beam. The spatial-spectral homogeneity map of the output beam at the exit of every CASCADE unit was measured with a 50-μm-diameter pinhole to sample the beam along the transverse direction, as shown in Fig. 3B. The homogeneity (Fig. 3C) was computed as defined in (*28*), revealing that the output spectrum has a calculated homogeneity value of 99.6% (99.5, 99.0, 98.7, and 93.5%) within the 1/*e*^2^ diameter of the beam at the input (first, second, third, and fourth units of CASCADE). Each measured result of spatial-spectral homogeneity showed quantitatively good agreement with the simulation result, and an ideal value of 95.6% was produced for the last unit. It is also worth mentioning that both experimental observations and numerical simulations indicate that there is a wavelength-dependent beam size or wavefront. Figure 3 (A and B) shows a considerable spatial-spectral variation close to a wavelength of 1030 nm. The spot sizes (beam divergence) of short wavelengths ranging from 450 to 800 nm are similar, but 40% smaller than those of long wavelengths ranging from 900 to 1300 nm on average. Fortunately, such wavelength-dependent beam size and spatial-spectral variation do not deteriorate the beam quality at the focus (inset in Fig. 1B) and lengthen the pulse duration (Fig. 2E), corroborated by theoretical simulations (section S1).

**Fig. 4.**
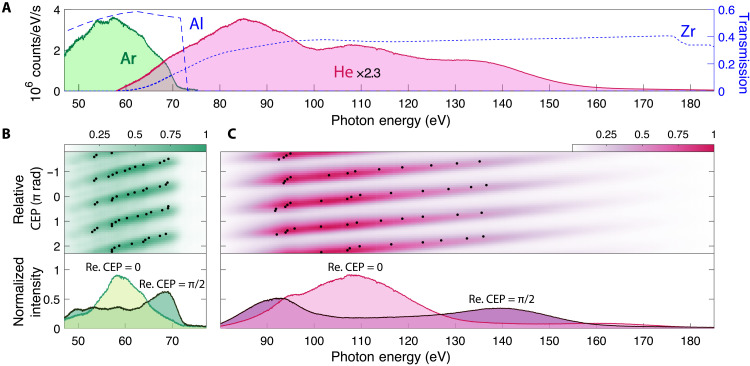
CEP-dependent HH spectra in Ar and He. (**A**) HH supercontinuum driven by the CASCADE single-cycle pulses in Ar and He, together with the transmission of filters. HH spectra in Ar (**B**) and He (**C**) acquired for varying CEP in steps of 250 mrad and with integration time of 10 s each. Black dots mark the peak of each HH spectrum, which vary approximately linearly with the CEP. A marked change of the HH spectrum for a relative CEP change of 0.5π rad is shown at the bottom.

The pulse duration and contrast of the postcompressed pulses from CASCADE were further investigated by conducting an HHG experiment to generate isolated attosecond pulses. The CEP stability of the resulting single-cycle pulses was measured to be 212 mrad using a single-shot *f*-to-2*f* interferometer (fig. S8). By using a concave mirror with 40-cm focal length, the pulse was focused into a semi-infinite gas cell filled with Ar or He (section S3 and fig. S9). Figure 4A shows a typical HH supercontinuum driven by CASCADE single-cycle pulses in Ar and He along with the transmission of the filters. The resulting HHG spectra are supercontinuous without modulations with the fundamental frequency, indicating that the driving pulse is short enough that the HHG emission is confined in the subcycle, which directly supports that optical energy has been well compressed by CASCADE. Notably, the generation of the HH supercontinuum using Ar is much more challenging than that using He, because weak fields can also ionize Ar, producing low-order harmonics. That is, the spectral features in the low-energy region can better reflect the temporal contrast of the driving pulse. The resulting HH EUV supercontinua in He covers photon energies from 60 to 180 eV. As illustrated in Fig. 4 (B and C), for the HH spectra in Ar and He recorded as a function of the relative CEP phase, these spectra repeat with π rad periodicity because there are two electric field peaks in one optical cycle. The bottom rows in Fig. 4 (B and C) further highlight a marked change in the HH spectrum for a relative CEP change of 0.5π rad. Such a high contrast of the CEP-dependent supercontinuum directly reflects the stability and temporal contrast of postcompressed pulses.

**Fig. 5.**
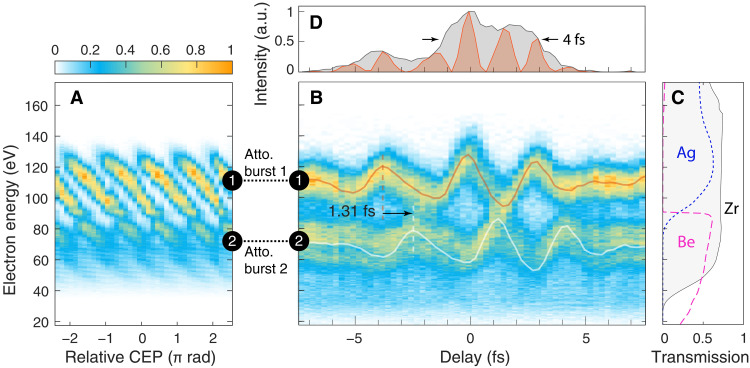
Attosecond streaking. (**A**) The CEP-dependent EUV (photoelectron) spectra obtained by ionizing Ne. With the CEP ≈2.3π rad set to maximize the HH energy from He, (**B**) the photoelectron streaking spectrogram, containing two traces at 110 and 70 eV, was obtained by varying the delay between the EUV pulse and the IR probe pulse. The vector potential of the IR probe pulse was extracted from the momentum of photoelectrons either at 110 eV (red line) or 70 eV (white line). The profiles of the two traces are nearly identical, but with a temporal separation of 1.31 fs equal to a half cycle of the driving laser field. (**C**) The transmission of 100-nm Zr (shown in gray area) used in the experiment. A short (long)–pass filter, e.g., 100-nm Ag (400-nm Be), can be applied to isolate the attosecond bursts at 110 eV (70 eV). Note that the axis of electron energy in (C) has been subtracted by the Ne photoionization potential (21.56 eV). (**D**) The square of the extracted vector potential (red area) and the associated envelope (black area) show an estimated duration of 4 fs in FWHM for the probe pulse.

Last, we conducted the attosecond streaking experiment to analyze the IR pulse from CASCADE and the resulting EUV pulses from He (see Materials and Methods for further details). By focusing EUV pulses into a Ne detection gas jet, Fig. 5A shows the dependence of generated photoelectron spectra on the CEP. With the fixed CEP ≈2.3π rad, the streaked photoelectron spectrum as a function of the delay between the EUV pulse and the IR probe pulse was measured as illustrated in Fig. 5B, which resolves the momentum shift of the photoelectron across the entire photoelectron spectrum. The two distinct streaking traces here verify the generation of two attosecond bursts at energies of 110 and 70 eV, respectively, with a temporal separation of 1.31 fs between them equal to a half cycle of the driving laser field, because these attosecond pulses are generated from subsequent half cycles. One can apply a short (long)–pass filter, e.g., Ag (Be) as shown in Fig. 5C, to isolate the attosecond pulses at 110 eV (70 eV) (*29*, *30*). For the pulse at 110 eV, the retrieved duration reaches 290 as (TL is 85 as) in FWHM (fig. S11). In addition, the vector potential of the IR probe pulse can be extracted from an attosecond streaking trace by tracking the evolution of the center of mass of the photoelectron spectrum over time (*11*), as shown in Fig. 5B. According to the square of the vector potential and the associated envelope profile of the IR probe pulse shown in Fig. 5D, the retrieved FWHM duration of ≈4 fs is close to the one acquired from the TIPTOE measurement shown in Fig. 2E. These streaking results confirm the capability of CASCADE for producing stable, millijoule-level pulses with a satisfactory beam quality and a single-cycle pulse duration to drive isolated attosecond pulses.

## DISCUSSION

Regarding gas selection in the current CASCADE, we found that Ar is the most appropriate gas for several reasons. Considering the ratio 2.3:1:0.08:0.04 for comparing nonlinear indices between Kr, Ar, Ne, and He, it has been found that using Kr or Ar facilitates the realization of a stronger self-phase modulation for spectral broadening at lower gas pressures than Ne and He (*31*). However, to inhibit the occurrence of self-trapping characterized by the critical power $P_{\text{cr}}=3.77\lambda_{0}^{2}/(8\pi n_{0}n_{2})$ (*32*), where λ_0_ is the vacuum wavelength of the driving laser and *n*_0_ is the linear refractive index, Ar represents the moderate choice to keep *P*_cr_ higher than the peak power of the laser pulse inside a CASCADE unit. In addition, the higher ionization potential of Ar compared to Kr tends to prevent significant ionization from occurring at the focus of a CASCADE cell. Last, apart from noble gases, molecular gases show great potential in postcompression, owing to the additional degree of freedom in vibration and rotation (*33*). However, heating problems could limit the scalability of molecule-based postcompression to a high repetition rate system with several tens of watts (*14*).

We used loose focusing geometry in CASCADE to avoid significant Ar ionization. According to the simulations, the peak intensity in the first, second, third, and fourth focus is estimated as 8.57 × 10^12^, 1.81 × 10^13^, 3.34 × 10^13^, and 1.66 × 10^14^ W/cm^2^ that produces 7.54 × 10^−21^, 1.06 × 10^−13^, 1.5 × 10^−9^, and 2.13 × 10^−3^ ionization level in Ar based on the Ammosov-Delone-Krainov ionization model (*34*), respectively. Ionization was negligible in the first three units of CASCADE, and self-phase modulation is the primary physical origin of supercontinuum generation. In the fourth unit, the increased ionization results in a sharp drop in the refractive index after the pulse peak and blueshifting spectrum at the trailing edge of the pulse (see section S1). Notably, CASCADE is obviously different from filaments, in which the balance of self-focusing compensates for the divergence of plasma, and diffraction repeats multiple times while maintaining the beam within a certain channel. In filaments, the temporal pulse profile varies considerably with the lateral position, and only the center region, which contains a small fraction of the full energy, can be used as short pulses (*14*).

We introduced a postcompression arrangement, CASCADE, to efficiently shorten pulses into the single-cycle regime. Implementing the four-unit CASCADE in Ar cells, we have compressed 1.34-mJ and CEP-stabilized pulses from 157 fs (50 cycles at 1030 nm) to 3.1 fs (single cycle at 885 nm), with an output of 0.98 mJ. Because pulse compression is provided immediately after each spectral broadening, CASCADE can efficiently shorten the pulse and markedly minimize reflections from mirrors, avoiding unwanted spectral and power losses. In HHG using single-cycle pulses, we observed a broad and highly CEP-dependent EUV continuum that provided stable isolated attosecond pulses. Thus, our postcompression method paves the way for the direct use of cost-effective, compact, and efficient Yb laser technologies in strong-field laser-driven experiments and attosecond science. CASCADE is scalable to higher pulse energies with a higher repetition rate. Another exciting prospect is the capability of CASCADE to efficiently shorten millijoule-level short-wavelength pulses, such as visible or ultraviolet light, which would produce highly intense HHG beams and isolated attosecond pulses at EUV and soft x-rays (*35*), offering a promising route to attosecond nonlinear spectroscopy (*36*) and attosecond pump probe spectroscopy (*37*).

## MATERIALS AND METHODS

### Temporal characterization—TIPTOE

The waveform was measured using TIPTOE to examine the temporal profile rigorously. A Mach-Zehnder–type interferometer was used in the experiment (see section S2). The laser beam was spatially divided into central and annular parts by a drilled mirror. To introduce a relative time delay, a piezoelectric stage was inserted into the interferometric setup. After spatial recombination of the two beams by another drilled mirror, the entire beam was directly focused in air. The intense annular beam ionized air at the focus, whereas the central beam perturbed the ionization yield. The modulation of the ionization yield, measured as a function of the time delay between the two beams, represents the electric field of the postcompressed pulse.

### Attosecond streaking

An attosecond streak camera was used, as shown in fig. S10, to analyze the CASCADE and resulting attosecond pulses. The IR pulse was split into two arms using a wedge. The first surface reflection of the wedge contained approximately 3.5% energy and served as the probe pulse. The transmitted IR beam was focused into a semi-infinite cell filled with He to generate EUV pulses. The EUV and residual fundamental were then passed through a customized filter with Zr foil at the center supported by a hole-drilled fused silica plate. The central filter blocks the residual fundamental, whereas the transmitted annular IR is used for time delay stabilization. Subsequently, the hole-drilled mirror was combined with the EUV beam with the probe beam. Both the EUV and probe beams are collinearly reflected by a SiO_2_ mirror and a Ni-coated elliptical mirror at a grazing angle of 6°. Through the elliptical mirror, both beams were focused into a Ne detection gas jet, while the EUV beam generated photoelectrons, which were collected using a 60-cm-long magnetic bottle electron time-of-flight spectrometer. Photoelectrons were recorded as a spectrogram with a time delay between the EUV and probe pulses. For feedback control, a piezoelectric transducer that controls and stabilizes the time delay between the EUV and the probe pulses and the interference fringes from 1030-nm light, spectrally selected by a narrow-bandwidth filter, were used.

## Supplementary Materials

### This PDF file includes:

Sections S1 to S3 Figs. S1 to S11 Table S1 References
