# Supplementary material for: Nonlinear compression toward high-energy single-cycle pulses by cascaded focus and compression
Source: Sci Adv. 2022 Aug 3;8(31):eabo1945. doi: 10.1126/sciadv.abo1945 (PMC9348793; doi:10.1126/sciadv.abo1945)
Supplement: Supplementary file 1 — Sections S1 to S3 Figs. S1 to S11 Table S1 References [file sciadv.abo1945_sm.pdf]

Science Advances
AAAS

Supplementary Materials for

Nonlinear compression toward high-energy single-cycle pulses by cascaded focus and compression

Ming-Shian Tsai *et al.*

Corresponding author: Ming-Chang Chen, mingchang@mx.nthu.edu.tw

Sci. Adv. **8**, eabo1945 (2022)
DOI: 10.1126/sciadv.abo1945

The PDF file includes:

Sections S1 to S3
Figs. S1 to S11
Table S1
Legend for movie S1
References

Other Supplementary Material for this manuscript includes the following:

Movie S1

Supplementary Text

S1. Simulation for spectral broadening in CASCADE

By implementing the split-step Fourier algorithm (*38*, *39*), a 3-D model was developed to simulate the spectral broadening and estimate the accumulated B-integral in each CASCADE unit. Here, the laser field $E(t,x,y,z)$ is defined with the temporal *t*-domain in the axial direction and the spatial *x* and *y* domains in the transverse directions, while *z* denotes the position of the field when it propagates inside a unit. Therefore, the associated field in the Fourier domain $E(\omega,k_x,k_y,z)$ is calculated using the angular frequency ω and wave vectors k_x and k_y relative to the *x* and *y* domains. With a transfer function,

$$H(\omega,k_x,k_y,dz) = \exp\left(i\sqrt{k(\omega)^2 - k_x^2 - k_y^2}dz\right), \quad \text{(S1)}$$

which accounts for the phase change of the field over an incremental distance *dz*, the diffraction and dispersion of field *E* when it propagates from *z* to *z* + *dz* are obtained according to

$$E'(\omega,k_x,k_y,z+dz) = E(\omega,k_x,k_y,z)\times H(\omega,k_x,k_y,dz), \quad \text{(S2)}$$

where the introduced wave vector $k(\omega) = \omega n(\omega)/c$ is a function of the refractive index $n(\omega)$ and the speed of light *c*. The following step involves transforming the intermediate field $E'(\omega,k_x,k_y,z+dz)$ to $E'(t,x,y,z+dz)$ and calculating the field $E(t,x,y,z+dz)$ at position *z* + *dz*

$$E(t,x,y,z+dz) = E'(t,x,y,z+dz)\times exp[ik_0\Delta nI'(t,x,y,z+dz)dz], \quad \text{(S3)}$$

which includes the nonlinear phase shift determined by the change in the refractive index Δn, the wave vector at central frequency k_0, and the intensity $I' = c\epsilon_0|E'|^2/2$ with the vacuum permittivity ϵ_0. Iterative calculations between Eqs. S2 and S3 can subsequently resolve the variation in field *E* throughout its propagation in a unit cell until it reaches its final destination. For the Ar gas utilized in this study, the linear refractive index n_0 for the propagation of each field component of wavelength λ_L was estimated using Cauchy's formula:

$$n_0(P_{opt},\lambda_L) = 1 + \frac{P_{opt}}{P_{atm}}A\left(1+\frac{B}{\lambda_L^2}\right), \quad \text{(S4)}$$

where P_{opt} is the operating gas pressure for a specific CASCADE unit, P_{atm} is the atmospheric pressure that gives A = 2.792 x 10^{-4} and B = 5600 nm^2 for Ar (*40*). In addition, the nonlinear change in the refractive index caused by the Kerr effect is calculated as

$$\Delta n_{Kerr} = \left(P_{opt}/P_{atm}\right) n_2 I[1-w_i], \quad \text{(S5)}$$

which is a function of the operating pressure P_{opt}, the reported value n_2 = 0.98 × 10^{-19} cm^2/W (*31*) for Ar at 1 atm, the pulse intensity I, and the ionization level w_i that was calculated only in the simulation of the 4th unit. The simulation of each CASCADE unit starts from the definition of the input pulse that resembles the experimental one in pulse energy, transverse profile, and spectral properties characterized by TIPTOE for the amplitude and spectral phase. The input pulse is focused by applying a quadratic phase in the space domain. With the t-cell size Δt =2 fs and x- and y-cell sizes Δx = Δy = 40 μm, the model dimensions of 512 × 256 × 256 were assigned in the time (t) and space (x, y) domains for simulating the 1st to 3rd units, while the applied Ar pressures were 680 torr, 640 torr, and 160 torr, respectively, to closely reproduce the results of spectral broadening observed in the experiments. These assigned gas pressures were subject to deviations of < 30 % relative to the experimental values (Table 2 in the main text), which can result from the uncertainty of the nonlinear index n_2 adopted in the simulations. As the laser intensity became > 5 ×10^{13} W/cm^2 around the focus of the 4th unit, the simulation implemented the Ammosov–Delone–Krainov (ADK) ionization model to estimate the density of the generated plasma electrons (*34*), and the associated change in the refractive index was calculated as follows:

$$\Delta n_{plasma} = -\frac{\lambda_0^2}{2\pi}P_{opt}N_0 r_e w_i, \quad \text{(S6)}$$

according to the central wavelength λ_0= 1030 nm, the operating gas pressure P_{opt}, the atom density N_0 = 3.3 × 10^{22}/m^3 for 1 torr at 20 ℃, the ionization level w_i calculated by the ADK model, and

the classical electron radius r_e= 2.818 × 10^{-15} m. To accommodate the reduced duration and expanded transverse profile of the pulse, the model size for the 4th unit is extended to 768 × 384 × 384 with the commensurate reduction of t-cell size to Δt = 1 fs, for which the simulated pulse propagation and spectral broadening with Ar of 240 torr that highly reproduce the experimentally measured spectrum are illustrated in Fig. S1.

Fig. S2 A illustrates the calculated ionization level of Ar that reached w_i = 2.13 × 10^{-3} when the focused pulse moved to the position of z = 2450 mm in the 4th unit with a peak intensity 1.66 × 10^{14} W/cm^2. Consequently, as shown in Fig. S2B, the increased electron density resulted in a sharp drop of the overall change in the refractive index $\Delta n = \Delta n_{Kerr} + \Delta n_{plasma}$ near the pulse peak and contributed to the enhancement of the blueshifting spectrum within the trailing edge of the pulse. By applying a lens function with a 2700-mm focal length to collimate the output pulse, Fig. S2C illustrates its spatiospectral phase distribution for identifying the wavefront deviations among the spectral components. The results indicate that the red components typically exhibit greater radius of curvature than those of the blue counterparts. Remarkably, a relatively uniform phase distribution enclosed within the transverse dimension of beam waist at the 1/e^2 level (cf. Fig. 3A) can be obtained through the majority of the spectral components, providing the possibility to realize a qualified focusing property for this spectrally broadened pulse. With such a wavelength-dependent beam size and wavefront, the simulation confirmed that the output beam of CASCADE can be refocused into a good beam profile with a single-cycle pulse duration that agrees well with the experimental results illustrated in Figs. 1B and 2E, respectively. The simulation also showed that using a partially closed iris aperture to select the central portion of the beam can consequently acquire an improved focusing quality for the beam in the subsequent applications, e.g., HHG (see Section S3).

To identify the propagation property of the pulse throughout the spectral broadening process, Fig. S2D compares the variation of the pulse transverse size when the 4th unit was assigned with the nonlinearity of both Δn_{Kerr} and Δn_{plasma} from 240-torr Ar, with the one of simply Δn_{Kerr} from 240-torr Ar, or with a vacuum. Comparing to the case of free space propagation, the presence of nonlinearity inherently reduces the beam size in the focal region. The contribution from Δn_{plasma} acts to inhibit the beam divergence after the focus as it cancels part of the accumulated phase change due to Δn_{Kerr}; however, the smooth diverges of the beam in the 4th unit (with Δn_{Kerr} and Δn_{plasma}) verifies that the mechanism of CASCADE can be distinguished from the filamentation in which a guided propagation of pulse over a certain distance is generally a prerequisite.

The gas pressure of 240 torr assigned to simulate the 4th unit is considerably higher than the experimental value of 150 torr, which can be attributed to the imperfection of the focus quality and/or gentle wavefront distortion, particularly accumulated among the waves around 1030 nm in the experiment, resulting in additional uncertainties to accurately reproduce the beam properties in the simulation. Nevertheless, based on the considerable agreement between the measured and simulated spectra as well as the consistency of the spatio-spectral beam homogeneity among them, these products indicate that self-phase modulation is the primary mechanism behind CASCADE in conjunction with a possible ionization level of ≈ 0.2 % in the last unit to achieve a significantly broadened spectrum.

S2. Waveform characterization by TIPTOE

A schematic of the TIPTOE experiment is shown in Fig. S3A (*25-27*). The laser pulse was spatially divided into two parts: the central beam and the outer beam, by a hole-drilled mirror. The central beam contained approximately 5 % intensity. The two beams passed through different optical paths to create a relative delay, and then recombined through another hole-drilled mirror. To monitor and precisely control the relative delay between the two pulses, we sent a narrowband diode laser at 405 nm into the setup from the rear side of one chirped mirror. The 405 nm laser went through the same

path as the single-cycle pulses and was then spatially separated by another chirp mirror. The relative delay between the outer and central beams was monitored by the 405 nm interference at a CMOS camera and locked in closed-loop mode by a piezo delay stage. The uncertainty in the relative delay is as low as ≈14 as, as shown in Fig. S3B.

The few-cycle pulse was focused into a gas cell filled with one-atmosphere air using a concave mirror with a focal length of 35 cm. The intense outer beam ionized the air at the focus, whereas the central beam perturbed the ionization yield. To avoid overionization in air, a partially closed iris was placed along the outer beam path. The ionization fluorescence was filtered by a short-pass filter with a wavelength shorter than 400 nm and then collected by a photomultiplier. A boxcar integrator was implemented to improve the signal by averaging 100 ionization events, with an electronic gate window of 10 μs. The modulation of the ionization yield, measured as a function of the time delay between the two pulses, represents the electric field of the pulse.

To confirm the validity of the TIPTOE measurement, a comparison was made between two individual experiments, characterizing the phase of the fused silica plates. Fig. S4 A-B presents the spectral phase of the original input pulses and the pulses dispersed after insertion of 250 μm (red) and 500 μm (blue) thick fused silica. The additional phases introduced by these two FS plates can be retrieved by subtracting their spectral phases. White light interferometry (WL) was applied to calibrate the phases of the two FS plates. Fig. S4 C shows that the phase curves match each other very well, directly confirming the validity of TIPTOE implemented in this study.

Interestingly, anywhere there is a spectral phase jump, the TIPTOE underestimates spectral intensity as presented in Fig. 2 and Fig. S4. The larger the phase jump, the lower the intensity. To understand this, we added a TIPTOE simulation using the ADK model, as presented in Fig. S5. The simulation was performed using a single-cycle pulse consisting of a smooth spectrum accompanied by two pronounced phase jumps close to approximately 650 nm and 900 nm for both the outer and central beams. At the focal plane, the outer pulse used in this simulation has a peak intensity of 1.55×10^{14} W/cm^2, while the peak intensity of the central pulse is 100 times weaker as 1.55×10^{12} W/cm^2. We calculated the ionization probability as a function of the time delay and performing the Fourier transform to obtain the spectrum. Fig. S5A shows the TIPTOE modulation signal. We found that the simulated TIPTOE signal (green dotted line) agrees well with the input pulse waveform (red line). Fig. S5B also shows that the phase retrieved from the TIPTOE (green dotted line) matched the original input phase (red line). However, there was a discrepancy between the spectrum retrieved from the TIPTOE (green area) and the original spectrum (red area). There is an underestimation of the spectral component close to the phase-jump wavelength (energy) at approximately 650 and 900 nm. This is because the group envelopes at ≈ 650 nm and ≈ 900 nm have been shifted to ≈ -12 fs and ≈ +50 fs, respectively (as presented in Fig. S5C), owing to the phase jump. A time window applied in this TIPTOE simulation will only sample a fraction of the group envelope at ≈ 650 nm and ≈ 900 nm, resulting in an intensity underestimation at these two wavelengths. Fortunately, this underestimation can be corrected using a wider time window in the TIPTOE measurements. Most remarkably, simulations reveal that regardless of the width of the sampling window, the TIPTOE always provides a correct waveform in the window, providing an accurate characterization of the pulse duration, as in the example shown in Fig. S5A.

S3. Generation of high-harmonic supercontinuum

The generation of a high-harmonic supercontinuum is the most straightforward and unambiguous way to distinguish the temporal contrast and CEP stability of few-cycle pulses. Fig. S6 shows the experimental setup used to produce HHG, which was driven by 3.1 fs and 665 μJ pulses at a central wavelength of 885 nm obtained by CASCADE, where the input beam diameter was 9 mm at the 1/e^2 level. The power stability of CASCADE pulses is shown in Fig. S7. The CEP stability of the single-cycle pulses was measured in a single-shot f-to-2f interferometer as 212 mrad, as shown in Fig. S8.

In HHG, the pulse is focused using a 40 cm concave mirror into either a semi-infinite gas cell (*41*) or a 2-mm-long cell. The gas cell was filled with Ar, Ne or He, which generated high harmonics. The semi-infinite cell consists of a gas-filled chamber with 0.2 mm thick Al foil. The harmonics exited through a hole drilled by the laser through the foil. In both geometries, to avoid overionization, a partially closed iris was placed along the laser beam path. The HHG yield was optimized by adjusting the iris size, gas pressure, and focus position near the cell. The pulse energy before and after the iris, corresponding iris size, and gas pressure after optimization are listed in Table S1. After the gas cell, the beam passes through 400 nm thick of either Al or Zr filters that block the residual fundamentals. The transmitted EUV beam was reflected by a concave EUV grating (600 lines/mm), resulting in a one-dimensional focus on the CCD camera plane. High-harmonic spectra were recorded using an X-ray CCD detector (Andor iKon-L CCD).

Fig. S9 shows the HHG spectra obtained for different gases and the two geometries. For comparison, the HHG spectra were acquired directly by reading the counts from an X-ray CCD detector (Andor iKon-L) without rescaling by the transmission of filters. Several interesting results were obtained.

I) In both geometries and all gas species, the resulting HHG spectra are supercontinuous without modulations with the fundamental frequency, indicating that the driving pulse is short enough that the HHG emission is confined in the sub-cycle and that the optical energy is well compressed by CASCADE.

II) We observed that the cutoff photon energy was higher when using a thin gas cell than when using a semi-infinite gas cell. For instance, in a semi-infinite gas cell, the cutoff photon energies in Ar, Ne, and He were 120 eV, 140 eV, and 180 eV, respectively. In the 2-mm-long cell, the cutoff photon energies in Ar and Ne were 130 eV and 170 eV, respectively. This is understandable because there is no plasma-induced defocusing before the driving pulse entering the thin gas cell compared with the semi-infinite gas cell. Consequently, the peak intensity in the thin gas cell could be higher in combination with defocusing-assisted phase matching (*42*), leading to a higher phase-matching cutoff.

III) We expect that in the thin cell geometry, the HHG from He can reach an even higher cutoff energy of 220-250 eV. Unfortunately, no harmonics were observed in He; even when the backing pressure increased to as high as ≈ 1.5 atm, the maximum pressure level that our current differential pump can handle. We speculate that the current gas cell geometry cannot withstand the high pressure required for a bright HHG. In addition, the thin gas cell suffers more reabsorption issues than the semi-infinite cell because of the higher ambient pressure. The pressure problem in the gas cell can be improved by using more turbomolecular pumps or multiple stages of differential pumping surrounding the target (*43*).

IV) Finally, when CASCADE compressed pulses from 157 fs to 3.1 fs, the central wavelength blueshifted from 1030 nm to 885 nm. Such a blueshift would slightly decrease the achievable photon energy because the phase-matching cutoff scaling is $\approx \lambda_0^{1.6-1.7}$, where λ_0 is the central wavelength of the pulse (*44*, *45*).

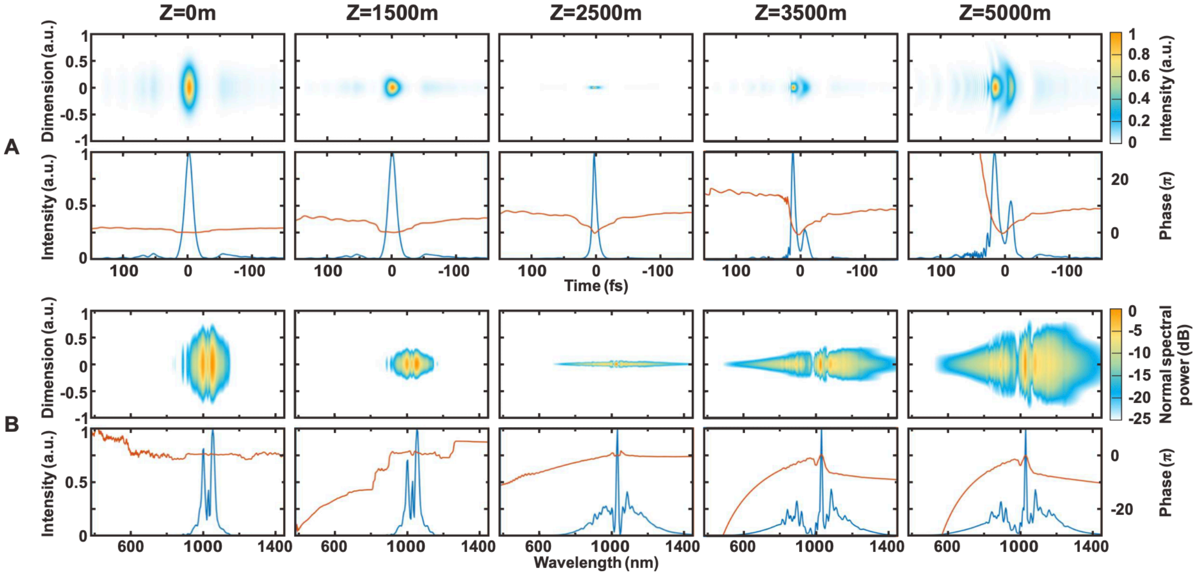

Fig. S1. Three-dimensional beam propagation simulation in the 4th unit of CASCADE. Simulated variations of (**A**) the spatial-temporal and (**B**) the spatial-spectral distribution of pulse throughout the spectral broadening (see also Movie S1). The corresponding on-axis temporal profile and spectrum with phases are provided below, respectively. The region of negative time corresponds to the leading edge of the pulse.

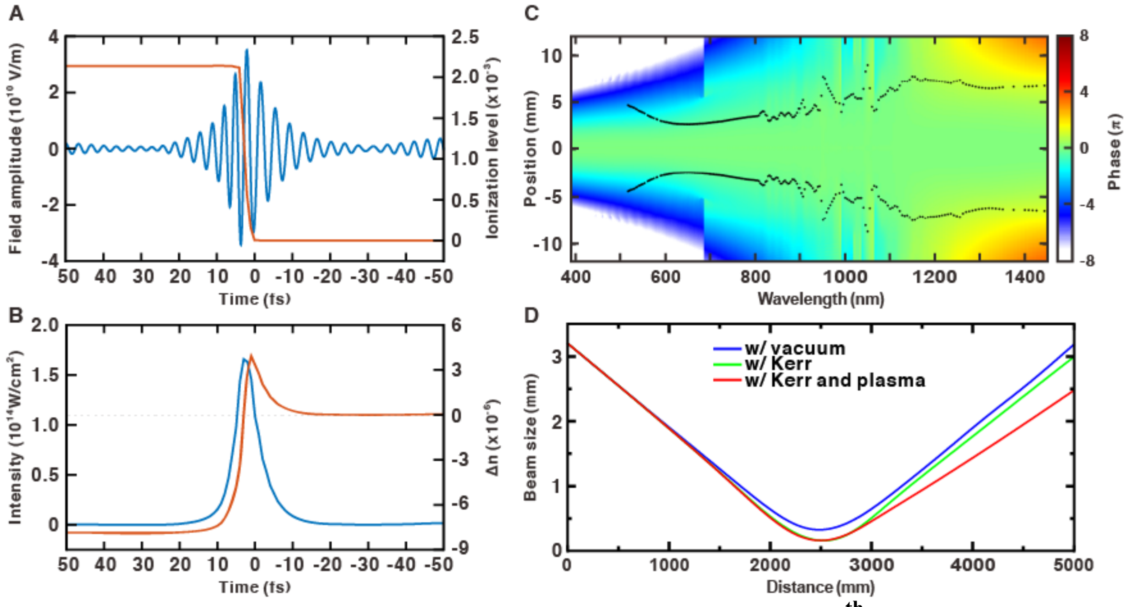

Fig. S2. Features of the spectral broadening simulation for the 4th unit of CASCADE. (**A**) Illustration of the laser field E_t and the calculated ionization level w_i for Ar when the pulse moved to the distance z = 2450 mm along with (**B**) the resulted overall change in the refractive index Δn with respect to the pulse envelop I. (**C**) The spatiospectral phase distribution of the collimated output pulse for comparing the wavefront deviations among the spectral components. The dotted lines in black indicate the beam waist at $1/e^2$ level (cf. Fig. 3). (**D**) The variation of the beam transverse size when the simulation is assigned with vacuum, the Kerr effect, or both the Kerr effect and plasma.

A

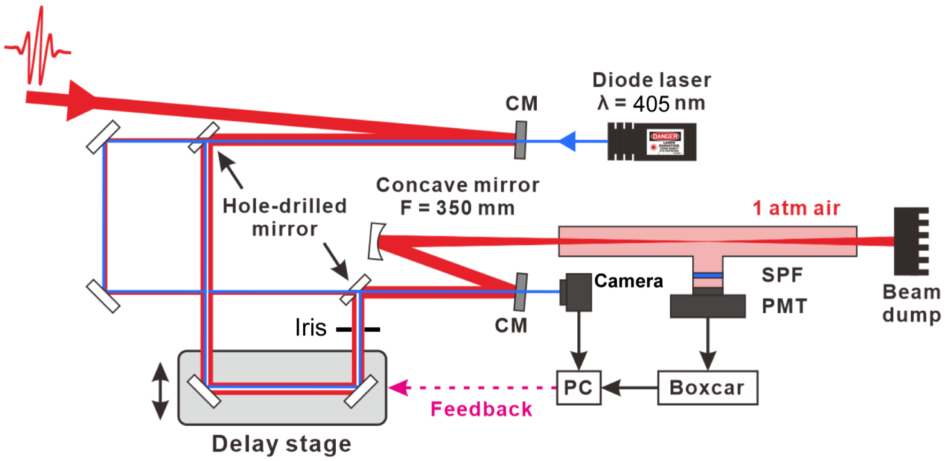

B

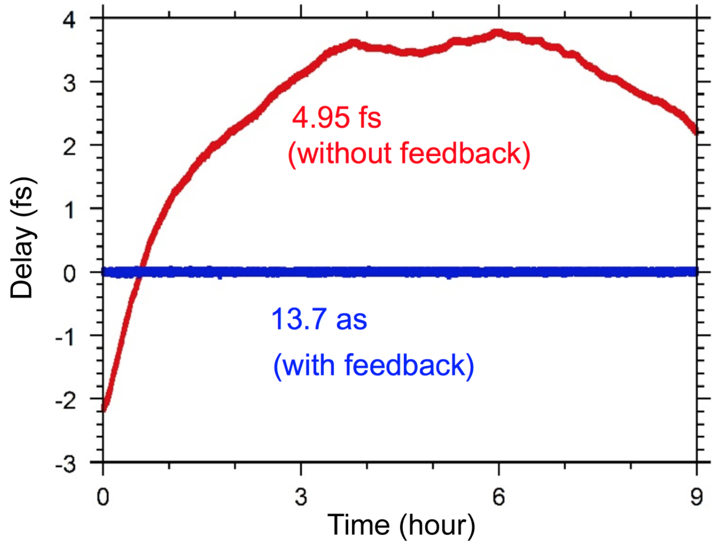

Fig. S3. Setup and delay stability of TIPTOE. (**A**) In our TIPTOE setup, a Mach–Zehnder-type interferometer was used. The laser beam was divided spatially into a central and an annular part by a drilled mirror. To introduce a relative time delay, a piezoelectric stage was inserted in one arm of the interferometric setup. After the spatial recombination of two beams by another drilled mirror, the entire beam was directly focused in air by one concave mirror. One 405 nm continuous laser was used to monitor and lock the delay between two arms. CM: chirped mirror; SPF: short-pass filter; PMT: photomultiplier; PC: personal computer. (**B**) The measured stability of the interferometer over 9 hours with (blue filled circle: 13.7 as rms) and without (red filled circle: 4.95 fs rms) the feedback loop.

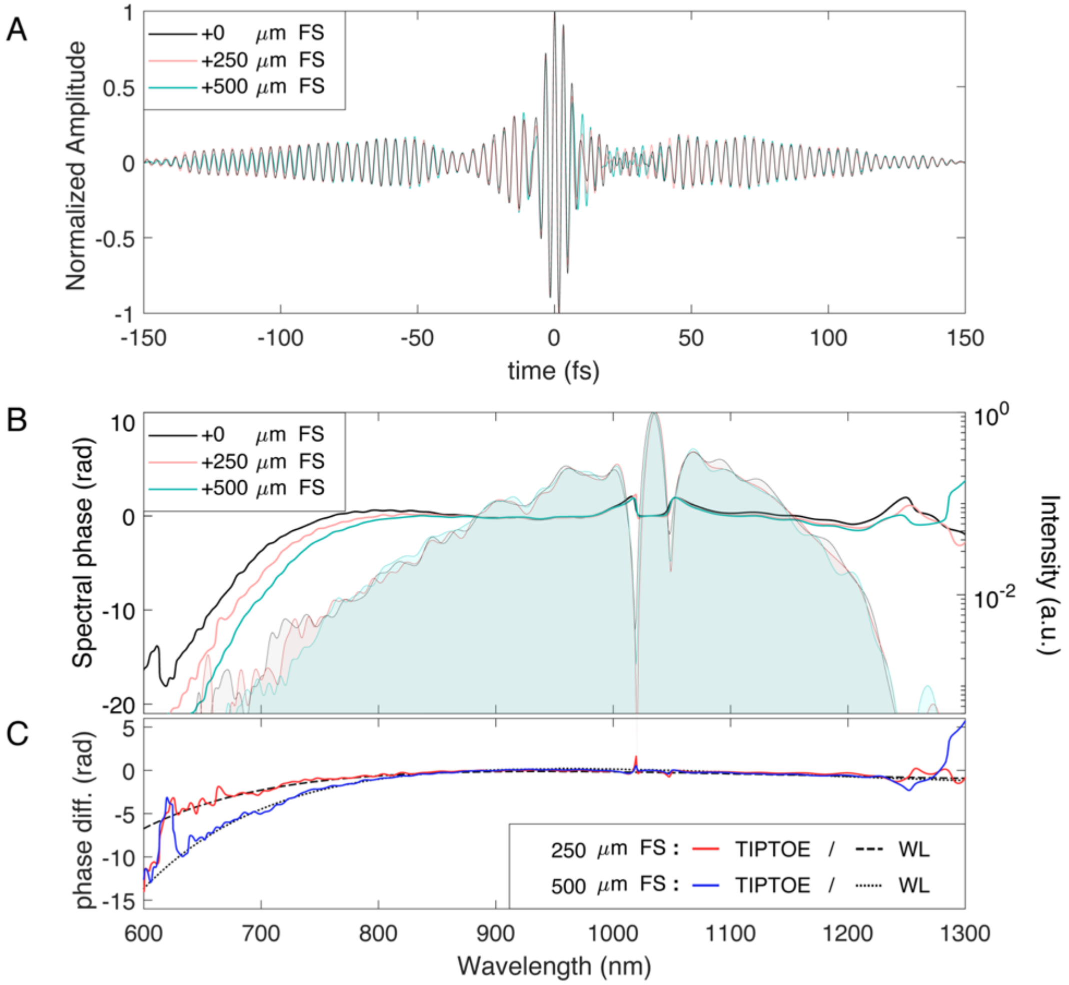

Fig. S4. Validity of the TIPTOE measurement. (**A**) Comparison of the temporal TIPTOE waveforms without (black) and with an additional insertion of 250 μm (red) and 500 μm (blue) thick fused silica (FS) plate. (**B**) The corresponding spectra and spectral phases were obtained by taking the Fourier transform of the measured waveform in (**A**). The three measurements show almost identical spectra, while short wavelengths undergo a dramatic retardance by the FS. (**C**) The phase introduced by these two FS plates can be retrieved by subtraction of their spectral phases. The phase of the two FS plates was also carefully calibrated by white light interferometry (WL). The excellent agreement in the FS phase between the TIPTOE and WL measurements confirms the validity of TIPTOE implemented in this study. In these TIPTOE measurements, the spectral bandpass filter ranging from 600 nm to 1300 nm was applied.

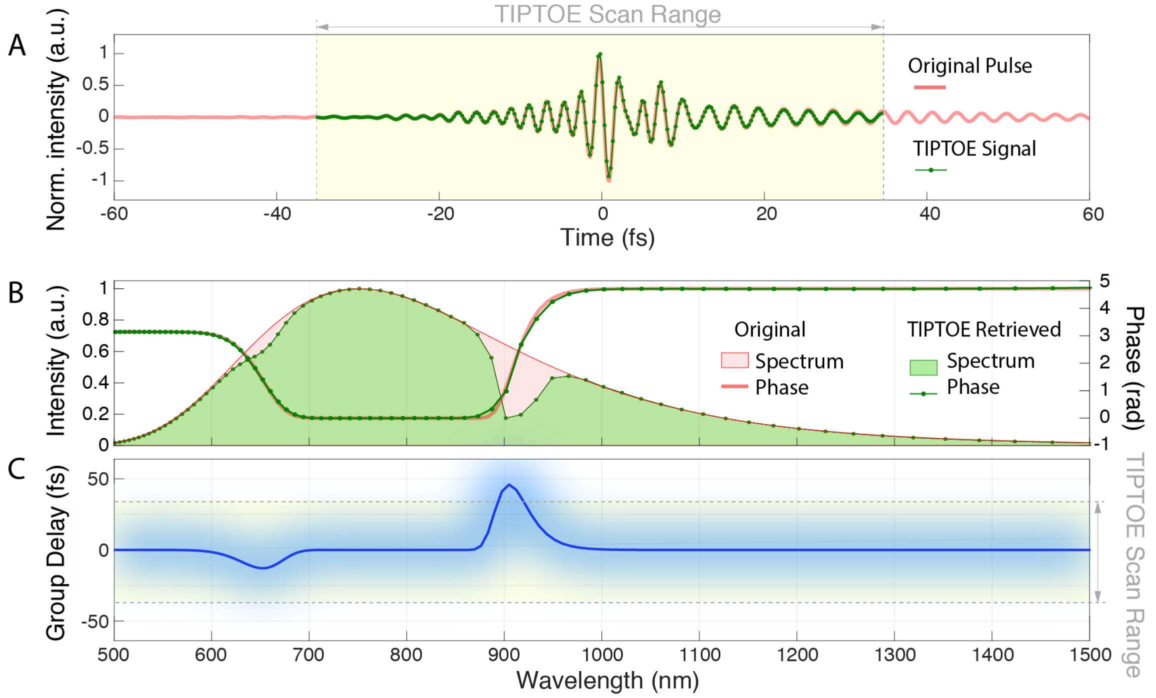

Fig. S5. Numerical Simulations of TIPTOE measurement. One numerical simulation calculated the ionization probability in Ar using ADK as a function of the time delay between the outer beam and the central beam, scanning from -35 fs to +35 fs and then performing the Fourier transform to obtain the spectrum. The simulation is performed using the same single-cycle pulse for the outer and central beams, which constituted a smooth spectrum accompanied by two pronounced phase jumps close to ≈ 650 nm and ≈ 900 nm. At the focus, the outer pulse used in this simulation has a peak intensity of 1.55×10^{14} W/cm^2, while the peak intensity of the central pulse is 100 times weaker at 1.55×10^{12} W/cm^2. (**A**) The simulated TIPTOE signal (green line with the sampling dots) agrees very well with the original waveform (red line). (**B**) The phase retrieved from the TIPTOE (green dotted line) also matches excellently with the original phase (red line). However, there is an underestimation of the spectral components close to the phase-jump wavelength (energy) at ≈ 650 nm and ≈ 900 nm. (**C**) The blue line represents the group delay of ≈ -12 fs and ≈ +50 fs experienced by the narrow-band light component at ≈ 650 nm and ≈ 900 nm, respectively. While the blue gradients highlight the duration of the group envelope, which is evaluated by the spectral resolution in (**B**) and based on the time-energy Fourier uncertainty relation. It is clear that a time window applied in (**A**) will only sample a fraction of the group envelope at ≈ 650 nm and ≈ 900 nm, resulting in an underestimation in the TIPTOE retrieved spectrum at these two wavelengths.

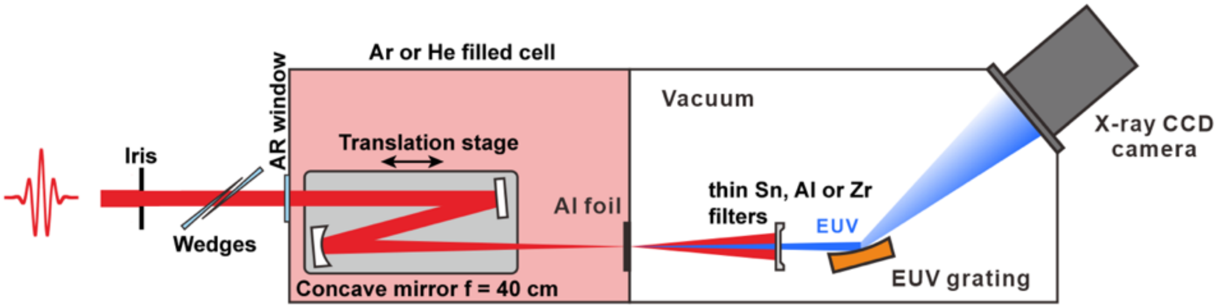

Fig. S6. Schematic of HHG in the semi-infinite cell. The cell consists of a gas-filled chamber with 0.2 mm thick Al foil. The gas cell is filled with Ar or He, where the high harmonics are generated. The harmonics exit through a hole drilled by the laser through the foil. To obtain optimal phase matching, the concave mirror was translated to precisely control the focal position near the Al foil (exit) of the cell. After passing through the semi-infinite cell, the HHG beam is diffracted by a concave EUV grating and then detected by an X-ray CCD camera. Metal filters are used to eliminate the fundamental laser light and to calibrate the spectrometer, depending on the photon energy range under investigation.

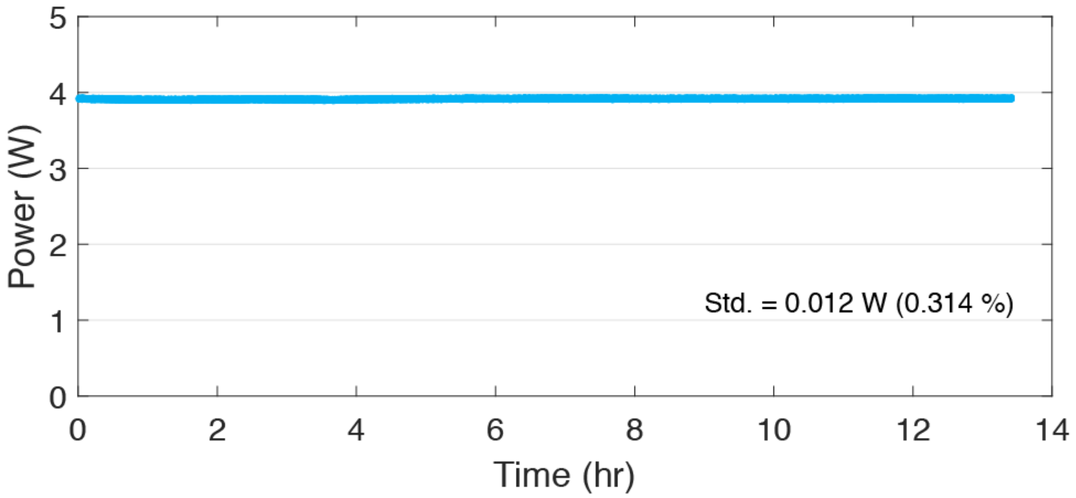

Fig. S7. Long-term output stability. Graph shows the 3.1 fs CASCADE pulse energy measured shot-by-shot and over a period of 13.5 hours. The average output power is 3.92 W (0.98 mJ @ 4 kHz) and the standard deviation is 0.013 W. Excellent stability, corresponding to 0.314 % rms, is obtained.

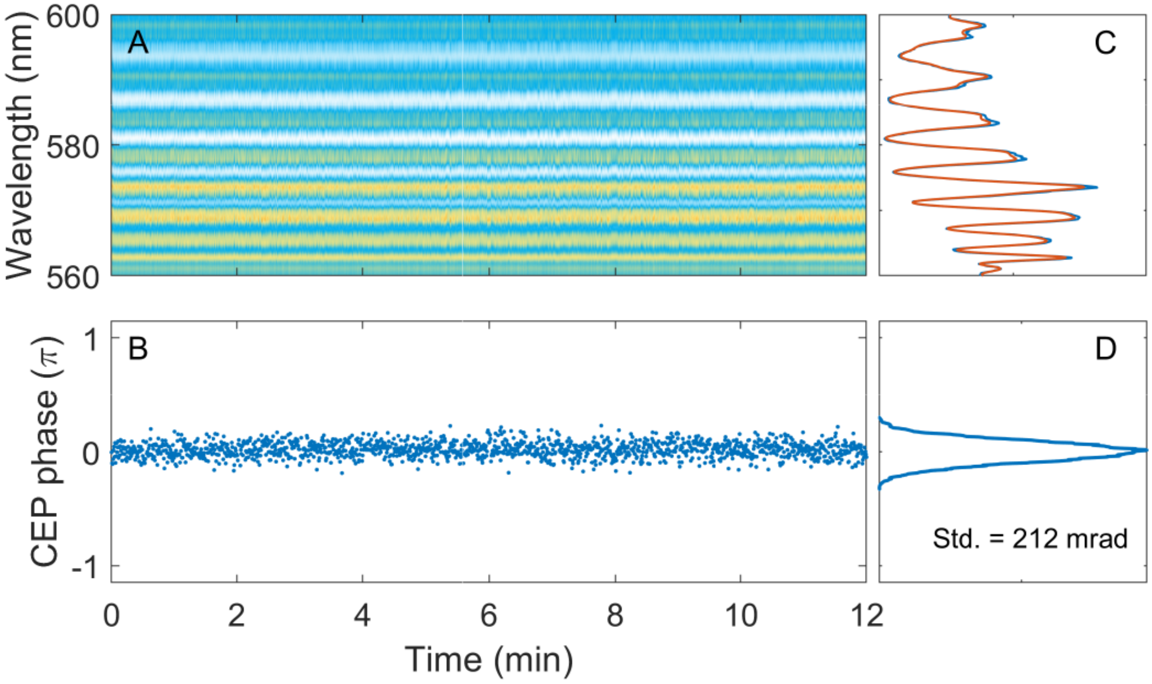

Fig. S8. Single-shot CEP of the 3.1 fs CASCADE pulse. The f – 2f spectrogram of the continuum (**A**) and the corresponding CEP (**B**) for over 12 minutes. (**C**) The single-shot (blue line) and the 100-shot-averaged (orange line) fringe patterns. (**D**) The histogram of the measured CEP in (**B**). The standard deviation of the CEP stability is 212 mrad. The CEP uncertainty is almost identical to that of the bulk continuum used in the front-end laser for the CEP stability loop. This indicates that CASCADE preserves CEP excellently in spectral broadening.

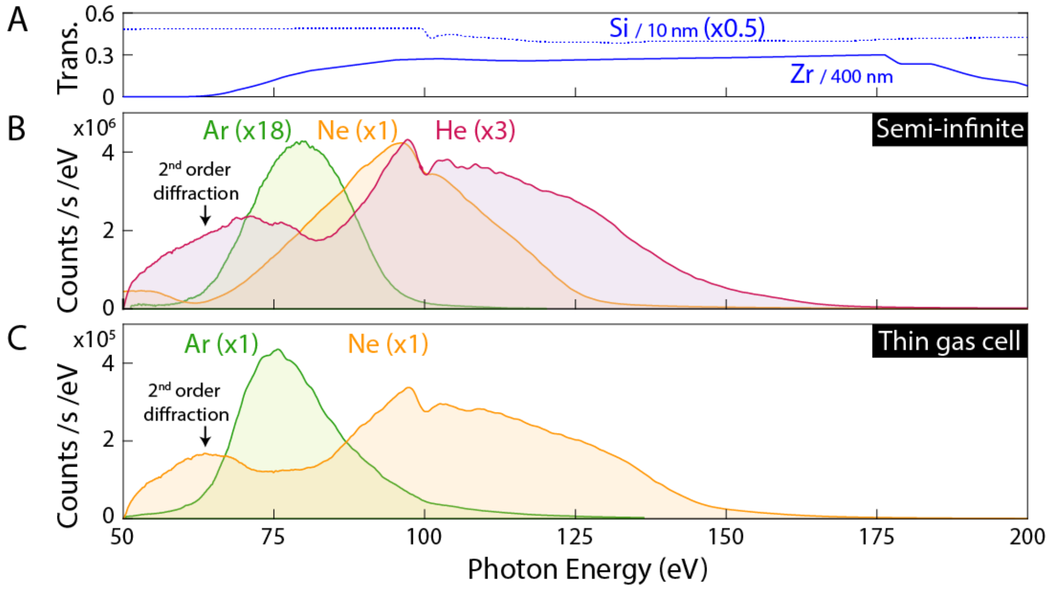

Fig. S9. HH supercontinuum in Ar, Ne, and He. (**A**) The filter transmission curves used to eliminate the driving laser light and calibrate photon energy. (**B**) The HH supercontinuum generated using a semi-infinite gas cell in Ar, Ne, and He. (**C**) The HH supercontinuum generated using a thin cell in Ar and Ne.

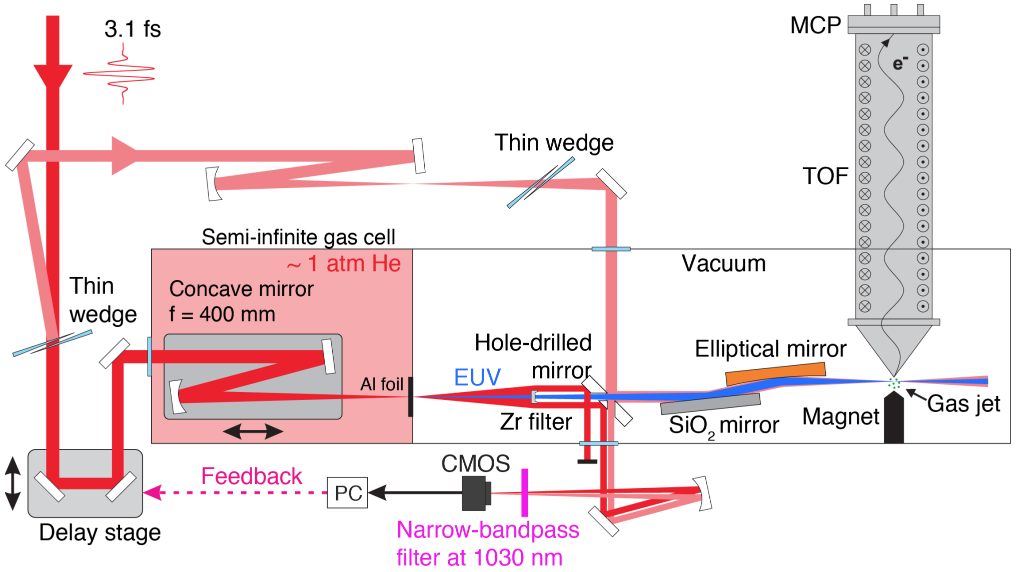

Fig. S10. Attosecond streaking. Schematic of the attosecond streaking camera. Attosecond pulses are created through HHG in a semi-infinite gas cell filled with He. The probe field is separated by a surface reflection from a thin wedge. Broadband reflection of the EUV supercontinuum is achieved through grazing-incidence reflection on one SiO_2 mirror and one Ni-coated elliptical mirror that focuses the EUV beam into the interaction region of a magnetic bottle time-of-flight electron spectrometer. One interferometer was used to monitor and lock the delay between the attosecond pulse and the probe field.

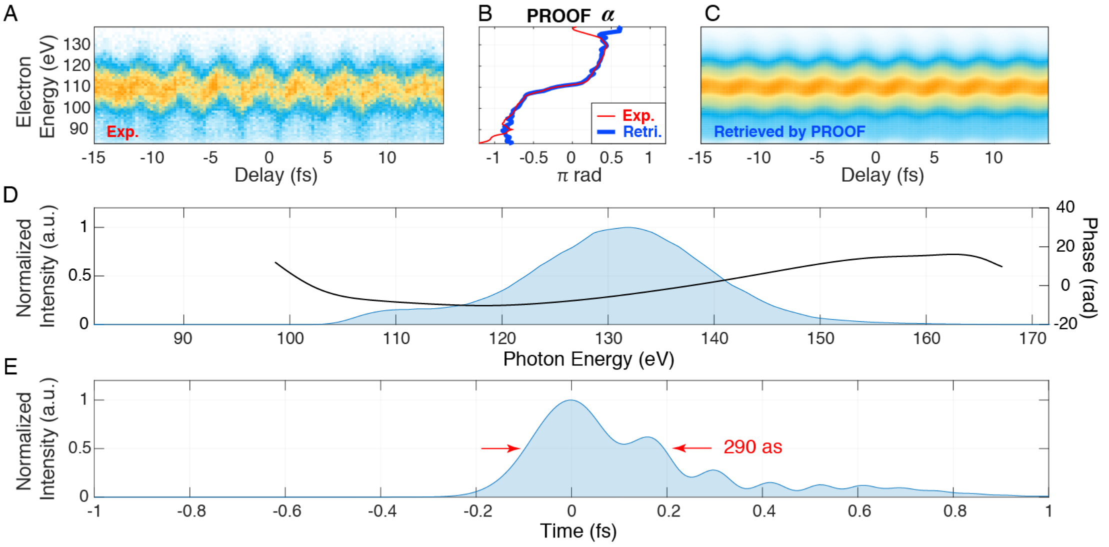

Fig. S11. Retrieval of the resulting attosecond pulse with PROOF. (**A**) Experimentally obtained laser-assisted photoemission spectrogram. Here a long and weak IR probe pulse was used for this spectrogram result. (**B**) A comparison of the phase angle *α* (see Ref.(*46*)) extracted from the experiment to that from the best retrieval shown in (**C**). (**D**) The PROOF-retrieved spectrum (blue area) and spectral phase (black line). The axis of electron energy in (**D**) has been added Ne photoionization potential (21.56 eV). (**E**) Retrieved temporal intensity profile shows a pulse duration of 290 as (TL is 85 as) in FWHM.

Table S1: Experimental parameters used in HHG.

Geometry	Gas	Actual pressure in the cell (torr)	Backing pressure (torr)	Input beam diameter (mm)	Optimal iris size (mm)	Focal spot size in vacuum‡ (μm)	Pulse energy before iris (μJ)	Pulse energy after iris (μJ)
S*	Ar	160	–	9.0	7.6	79	665	363
S*	Ne	420	–	9.0	8.8	72	665	400
S*	He	1082	–	9.0	9.9	68	665	465
T†	Ar	–	760	9.0	10.1	64	665	483
T†	Ne	–	1489	9.0	8.2	74	665	338

* S: Semi-infinite gas cell geometry
†T: Thin cell geometry (inner diameter is 2 mm.)
‡ Spot size in diameter

Movie S1.
The simulated evolution of the pulse profile and spectrum in the 4th unit of CASCADE.

REFERENCES AND NOTES

1. J. Li, J. Lu, A. Chew, S. Han, J. Li, Y. Wu, H. Wang, S. Ghimire, Z. Chang, Attosecond science based on high harmonic generation from gases and solids. *Nat. Commun.* **11**, 2748 (2020).

2. P. M. Kraus, B. Mignolet, D. Baykusheva, A. Rupenyan, L. Horný, E. F. Penka, G. Grassi, O. I. Tolstikhin, J. Schneider, F. Jensen, L. B. Madsen, A. D. Bandrauk, F. Remacle, H. J. Wörner, Measurement and laser control of attosecond charge migration in ionized iodoacetylene, *Science* **350**, 790–795 (2015).

3. E. Goulielmakis, Z.-H. Loh, A. Wirth, R. Santra, N. Rohringer, V. S. Yakovlev, S. Zherebtsov, T. Pfeifer, A. M. Azzeer, M. F. Kling, S. R. Leone, F. Krausz, Real-time observation of valence electron motion, *Nature* **466**, 739–743 (2010).

4. F. Krausz, M. I. Stockman, Attosecond metrology: From electron capture to future signal processing. *Nat. Photonics* **8**, 205–213 (2014).

5. H. Mashiko, Y. Chisuga, I. Katayama, K. Oguri, H. Masuda, J. Takeda, H. Gotoh, Multi-petahertz electron interference in Cr:Al_2O_3 solid-state material. *Nat. Commun.* **9**, 1468 (2018).

6. R. Géneaux, C. J. Kaplan, L. Yue, A. D. Ross, J. E. Bækhøj, P. M. Kraus, H.-T. Chang, A. Guggenmos, M.-Y. Huang, M. Zürch, K. J. Schafer, D. M. Neumark, M. B. Gaarde, S. R. Leone, Attosecond time-domain measurement of core-level-exciton decay in magnesium oxide. *Phys. Rev. Lett.* **124**, 207401 (2020).

7. Á. Jiménez-Galan, R. E. F. Silva, O. Smirnova, M. Ivanov, Lightwave control of topological properties in 2D materials for sub-cycle and non-resonant valley manipulation. *Nat. Photonics* **14**, 728–732 (2020).

8. F. Siegrist, J. A. Gessner, M. Ossiander, C. Denker, Y.-P. Chang, M. C. Schröder, A. Guggenmos, Y. Cui, J. Walowski, U. Martens, J. K. Dewhurst, U. Kleineberg, M. Münzenberg, S. Sharma, M. Schultze, Light-wave dynamic control of magnetism. *Nature* **571**, 240–244 (2019).

9. F. J. Furch, T. Witting, A. Giree, C. Luan, F. Schell, G. Arisholm, C. P. Schulz, M. J. J. Vrakking, CEP-stable few-cycle pulses with more than 190 μJ of energy at 100 kHz from a noncollinear optical parametric amplifier. *Opt. Lett.* **42**, 2495–2498 (2017).

10. N. Ishii, K. Kaneshima, K. Kitano, T. Kanai, S. Watanabe, J. Itatani, Sub-two-cycle, carrier-envelope phase-stable, intense optical pulses at 1.6 μm from a BiB_3O_6 optical parametric chirped-pulse amplifier. *Opt. Lett.* **37**, 4182–4184 (2012).

11. G. M. Rossi, R. E. Mainz, Y. Yang, F. Scheiba, M. A. Silva-Toledo, S.-H. Chia, P. D. Keathley, S. Fang, O. D. Mücke, C. Manzoni, G. Cerullo, G. Cirmi, F. X. Kärtner, Sub-cycle millijoule-level parametric waveform synthesizer for attosecond science. *Nat. Photonics* **14**, 629–635 (2020).

12. G. Krauss, S. Lohss, T. Hanke, A. Sell, S. Eggert, R. Huber, A. Leitenstorfer, Synthesis of a single cycle of light with compact erbium-doped fibre technology. *Nat. Photonics* **4**, 33 (2010), 36.

13. O. Pronin, M. Seidel, F. Lücking, J. Brons, E. Fedulova, M. Trubetskov, V. Pervak, A. Apolonski, Th. Udem, F. Krausz, High-power multi-megahertz source of waveform-stabilized few-cycle light. *Nat. Commun.* **6**, 6988 (2015).

14. T. Nagy, P. Simon, L. Veisz, High-energy few-cycle pulses: Post-compression techniques. *Adv. Phys.: X* **6**, 1845795 (2021).

15. M. Nisoli, S. De Silvestri, O. Svelto, Generation of high energy 10 fs pulses by a new pulse compression technique, *Appl. Phys. Lett.* **68**, 2793–2795 (1996).

16. Y.-G. Jeong, R. Piccoli, D. Ferachou, V. Cardin, M. Chini, S. Hädrich, J. Limpert, R. Morandotti, F. Légaré, B. E. Schmidt, L. Razzari, Direct compression of 170-fs 50-cycle pulses down to 1.5 cycles with 70% transmission. *Sci. Rep.* **8**, 11794 (2018).

17. R. Klas, W. Eschen, A. Kirsche, J. Rothhardt, J. Limpert, Generation of coherent broadband high photon flux continua in the XUV with a sub-two-cycle fiber laser. *Opt. Express* **28**, 6188–6196 (2020).

18. C.-H. Lu, Y.-J. Tsou, H.-Y. Chen, B.-H. Chen, Y.-C. Cheng, S.-D. Yang, M.-C. Chen, C.-C. Hsu, A. H. Kung, Generation of intense supercontinuum in condensed media. *Optica* **1**, 400–406 (2014).

19. R. Budriūnas, D. Kučinskas, A. Varanavičius, High-energy continuum generation in an array of thin plates pumped by tunable femtosecond IR pulses. *Appl. Phys. B* **123**, 212 (2017).

20. C.-H. Lu, W.-H. Wu, S.-H. Kuo, J.-Y. Guo, M.-C. Chen, S.-D. Yang, A. H. Kung, Greater than 50 times compression of 1030 nm Yb:KGW laser pulses to single-cycle duration. *Opt. Express* **27**, 15638–15648 (2019).

21. B. Zhu, Z. Fu, Y. Chen, S. Peng, C. Jin, G. Fan, S. Zhang, S. Wang, H. Ru, C. Tian, Y. Wang, H. Kapteyn, M. Murnane, Z. Tao, Spatially homogeneous few-cycle compression of Yb lasers via all-solid-state free-space soliton management. *Opt. Express* **30**, 2918–2932 (2022).

22. L. Lavenu, M. Natile, F. Guichard, Y. Zaouter, X. Delen, M. Hanna, E. Mottay, P. Georges, Nonlinear pulse compression based on a gas-filled multipass cell. *Opt. Lett.* **43**, 2252–2255 (2018).

23. M. Müller, J. Buldt, H. Stark, C. Grebing, J. Limpert, Multipass cell for high-power few-cycle compression. *Opt. Lett.* **46**, 2678–2681 (2021).

24. N. Raabe, T. Feng, T. Witting, A. Demircan, C. Brée, G. Steinmeyer, Role of intrapulse coherence in carrier-envelope phase stabilization. *Phys. Rev. Lett.* **119**, 123901 (2017).

25. S. B. Park, K. Kim, W. Cho, S. I. Hwang, I. Ivanov, C. H. Nam, K. T. Kim, Direct sampling of a light wave in air. *Optica* **5**, 402–408 (2018).

26. N. Saito, N. Ishii, T. Kanai, J. Itatani, All-optical characterization of the two-dimensional waveform and the Gouy phase of an infrared pulse based on plasma fluorescence of gas. *Opt. Express* **26**, 24591–24601 (2018).

27. Y. Liu, S. Gholam-Mirzaei, J. E. Beetar, J. Nesper, A. Yousif, M. Nrisimhamurty, M. Chini, All-optical sampling of few-cycle infrared pulses using tunneling in a solid. *Photonics Res.* **9**, 929–936 (2021).

28. J. Weitenberg, A. Vernaleken, J. Schulte, A. Ozawa, T. Sartorius, V. Pervak, H.-D. Hoffmann, T. Udem, P. Russbüldt, T. W. Hänsch, Multi-pass-cell-based nonlinear pulse compression to 115 Fs at 7.5 μJ pulse energy and 300 W average power. *Opt. Express* **25**, 20502–20510 (2017).

29. A. Baltuška, Th. Udem, M. Uiberacker, M. Hentschel, E. Goulielmakis, Ch. Gohle, R. Holzwarth, V. S. Yakovlev, A. Scrinzi, T. W. Hänsch, F. Krausz, Attosecond control of electronic processes by intense light fields. *Nature* **421**, 611–615 (2003).

30. M. Chini, K. Zhao, Z. Chang, The generation, characterization and applications of broadband isolated attosecond pulses. *Nat. Photonics* **8**, 178–186 (2014).

31. H. J. Lehmeier, W. Leupacher, A. Penzkofer, Nonresonant third order hyperpolarizability of rare gases and N_2 determined by third harmonic generation. *Opt. Commun.* **56**, 67–72 (1985).

32. G. Fibich, A. L. Gaeta, Critical power for self-focusing in bulk media and in hollow waveguides. *Opt. Lett.* **25**, 335–337 (2000).

33. J. E. Beetar, M. Nrisimhamurty, T.-C. Truong, G. C. Nagar, Y. Liu, J. Nesper, O. Suarez, F. Rivas, Y. Wu, B. Shim, M. Chini, Multioctave supercontinuum generation and frequency conversion based on rotational nonlinearity. *Sci. Adv.* **6**, eabb5375 (2020).

34. M. V. Ammosov, N. B. Delone, V. P. Karinov, Tunnel ionization of complex atoms and of atomic ions in an altemating electromagnetic field. *Sov. Phys. JETP* **64**, 1191 (1987).

35. D. Popmintchev, C. Hernández-García, F. Dollar, C. Mancuso, J. A. Pérez-Hernández, M.-C. Chen, A. Hankla, X. Gao, B. Shim, A. L. Gaeta, M. Tarazkar, D. A. Romanov, R. J. Levis, J. A. Gaffney, M. Foord, S. B. Libby, A. Jaron-Becker, A. Becker, L. Plaja, M. M. Murnane, H. C. Kapteyn, T. Popmintchev, Ultraviolet surprise: Efficient soft x-ray high-harmonic generation in multiply ionized plasmas. *Science* **350**, 1225–1231 (2015).

36. T. Helk, E. Berger, S. Jamnuch, L. Hoffmann, A. Kabacinski, J. Gautier, F. Tissandier, J.-P. Goddet, H.-T. Chang, J. Oh, C. D. Pemmaraju, T. A. Pascal, S. Sebban, C. Spielmann, M. Zuerch, Table-top extreme ultraviolet second harmonic generation. *Sci. Adv.* **7**, eabe2265 (2021).

37. Y. Kissin, M. Ruberti, P. Kolorenč, V. Averbukh, Attosecond pump–attosecond probe spectroscopy of Auger decay. *Phys. Chem. Chem. Phys.* **23**, 12376–12386 (2021).

38. M. Hanna, X. Délen, L. Lavenu, F. Guichard, Y. Zaouter, F. Druon, P. Georges, Nonlinear temporal compression in multipass cells: Theory. *J. Opt. Soc. Am. B.* **34**, 1340–1347 (2017).

39. R. Deiterding, R. Glowinski, H. Oliver, S. Poole, A reliable split-step fourier method for the propagation equation of ultra-fast pulses in single-mode optical fibers. *J. Lightwave Technol.* **31**, 2008–2017 (2013).

40. M. Born, E. Wolf, in *Principle of Optics* (Cambridge Univ. Press, ed. 7, 1999), pp. 100–101.

41. J. R. Sutherland, E. L. Christensen, N. D. Powers, S. E. Rhynard, J. C. Painter, J. Peatross, High harmonic generation in a semi-infinite gas cell. *Opt. Express* **12**, 4430–4436 (2004).

42. H.-W. Sun, P.-C. Huang, Y.-H. Tzeng, J.-T. Huang, C. D. Lin, C. Jin, M.-C. Chen, Extended phase matching of high harmonic generation by plasma-induced defocusing. *Optica* **4**, 976–981 (2017).

43. A. S. Johnson, D. R. Austin, D. A. Wood, C. Brahms, A. Gregory, K. B. Holzner, S. Jarosch, E. W. Larsen, S. Parker, C. S. Strüber, P. Ye, J. W. G. Tisch, J. P. Marangos, High-flux soft x-ray harmonic generation from ionization-shaped few-cycle laser pulses. *Sci. Adv.*. **4**, eaar3761 (2018).

44. T. Popmintchev, M.-C. Chen, A. Bahabad, M. Gerrity, P. Sidorenko, O. Cohen, I. P. Christov, M. M. Murnane, H. C. Kapteyn, Phase matching of high harmonic generation in the soft and hard x-ray regions of the spectrum. *Proc. Natl. Acad. Sci. U.S.A.* **106**, 10516–10521 (2009).

45. M.-C. Chen, P. Arpin, T. Popmintchev, M. Gerrity, B. Zhang, M. Seaberg, D. Popmintchev, M. M. Murnane, H. C. Kapteyn, Bright, coherent, ultrafast soft x-ray harmonics spanning the water window from a tabletop light source. *Phys. Rev. Lett.* **105**, 173901 (2010).

46. M. Chini, S. Gilbertson, S. D. Khan, Z. Chang, Characterizing ultrabroadband attosecond lasers. *Opt. Express* **18**, 13006–13016 (2010).
